# Pancreatic Cancer Risk in Hereditary Chronic Pancreatitis

**DOI:** 10.1016/j.gastha.2026.100889

**Published:** 2026-01-29

**Authors:** Hans Scherübl

**Affiliations:** Department Gastroenterology, Infectious Diseases and Rheumatology, Charité-Campus Benjamin Franklin, Berlin, Germany

Dear Editor,

I appreciate the excellent work by Diwan et al^1^ examining mild dilatation of the main pancreatic duct as a risk factor for progression to pancreatic cancer in high-risk individuals (HRIs). Diwan et al^1^ screened individuals at high risk for familial or hereditary pancreatic ductal adenocarcinoma (PDAC), and similarly, they followed up HRI suffering from hereditary chronic pancreatitis (HCP). As dilatation of the main pancreatic duct is a common sign in chronic pancreatitis,^2^^,^^3^ it would be interesting to know if mild dilatation of the main pancreatic duct was also forecasting progression to PDAC in carriers of HCP genetic mutations. Was dilatation of the main pancreatic duct observed with the same frequency, with the same pattern, and to the same extent in HCP patients as in HRI of hereditary PDAC?

Current guidelines recommend initiating PDAC surveillance in HCP patients at age 40 or 20 years after the first pancreatitis episode, whichever is earlier.^4^^,^^5^ The evaluation of biomarker signatures has recently been shown to facilitate early-stage PDAC detection in high-risk groups.^6^ Thus, the combination of pancreatic imaging with biomarker monitoring might become the backbone of future PDAC surveillance programs in HCP patients. Controlled prospective studies on the effectiveness of PDAC surveillance are still missing for HCP patients; they are much needed.

I very much appreciate the authors’ significant contribution to early PDAC detection in high-risk groups and would like to encourage further research incorporating these considerations.
